# Student conceptions of the production of cow's milk—An exploratory interview study with 6th- and 10th-grade students

**DOI:** 10.3389/fnut.2023.1112183

**Published:** 2023-04-06

**Authors:** Lena Szczepanski, Florian Fiebelkorn, Gesa Ostermann, Lisa Altevogt, Elena Folsche

**Affiliations:** Biology Didactics, Department of Biology/Chemistry, Osnabrück University, Osnabrück, Germany

**Keywords:** agriculture, dairy cow, milk, student conceptions, education for sustainable development (ESD), Germany

## Abstract

The production of food and the associated livestock farming contribute significantly to climate change and the global loss of biodiversity, hindering the achievement of the United Nations Sustainable Development Goals (SDGs). To promote responsible consumption and production of food (SDG 12), ensuring that students understand the production of our food, the associated livestock farming, and the interrelatedness of production and consumption is essential. Thus, Education for Sustainable Development (ESD) is an important tool for achieving the SDGs. To develop effective teaching and learning strategies to educate students about the production of food from livestock, it is important to identify students' existing conceptions of this topic. Thus, this study examined sixth-grade (*n* = 4; *M*_*Age*_ = 12 years; *SD*_*Age*_ = 0.7 years; 50% female) and tenth-grade students' (*n* = 4; *M*_*Age*_ = 16 years; *SD*_*Age*_ = 0 years; 50% female) conceptions of milk production, focusing on dairy farming, the milking process and techniques, and the production of cow's milk. Semi-structured interviews were conducted with students from Osnabrück (Lower Saxony) to elicit student conceptions. The evaluation of the students' conceptions was carried out using qualitative content analysis. The results largely indicated that both sixth and tenth graders had realistic conceptions of dairy farming and the milking process and techniques. However, some students also expressed romanticized conceptions of pasture grazing and calf rearing. In addition, unrealistic statements regarding the formation of milk were identified. The conceptions of the sixth and tenth graders were compared, and with a few exceptions, no significant differences were found between the two cohorts. However, the tenth graders tended to have more differentiated conceptions about milk production than the sixth graders. In conducting the analysis, it became clear that students' conceptions of the production of milk are influenced by individual primary experiences with dairy farms. Finally, based on these results, educational recommendations for the school teaching framework in the context of ESD and implications for further research are presented.

## 1. Introduction

Climate change and loss of biodiversity are caused to a considerable extent by the production of our food as well as our eating habits (1–5). Husbandry of farm animals, such as cattle for meat and milk production, is a main factor responsible for the current global environmental issues (2, 4). Approximately one-fifth of global anthropogenic greenhouse gases come from the agricultural sector (5). Around 80% of this is due to livestock farming, which accounts for 9% of global CO_2_ emissions and 40% of global methane emissions (4).

Germany is the largest cow's milk producer in the European Union, with approximately four million dairy cows and 33.1 million tons of cow's milk produced in 2019 (6, 7). Globally, Germany ranks third in imports and exports of agricultural goods (8). In 2019, approximately 16.7 million hectares of arable land were cultivated (9). Ninety percent of the farms in Germany specialized in one branch of agricultural production, such as arable farming or pig fattening. Approximately 69,000 farms were dairy farms (6, 10). In 2019, 45.5 billion euros were generated in Germany with agricultural products. Milk comprised 24% of the total product sales, with 11.1 billion euros in sales (11).

To keep up with this, farms in Germany have undergone a fundamental structural change in recent decades that is often referred to as “agricultural structural change” (6). Farms with livestock were particularly affected. As a result of increasing mechanization and specialization of farms focusing on certain products, livestock, and feed, the number of farms decreased steadily from 1.15 million in 1970 to 262,780 in 2020 (6, 12). In parallel, the number of people employed in the agricultural sector also decreased from 24.6% (1950) to 1.3% (2019) (13). In contrast, milk yield per cow increased from 2,480 kg (1950) to 8,457 kg (2020) per year (6, 12).

Due to these developments, there is an urgent need for action concerning various environmental burdens and the consequences of livestock farming. Agriculture—and with it, the agricultural and food system—needs restructuring with the goal of sustainable nutrition. This mainly affects dairy farming, one of the largest branches of agriculture (6). To bring about changes promoting sustainable nutrition, society's attitudes toward milk and dairy products need to be educated. It is essential to focus mainly on children and adolescents because eating behavior already manifests itself at these stages of life and is difficult to influence in adulthood (14, 15). Therefore, a central role in promoting sustainable nutrition is played by Education for Sustainable Development (ESD), which aims to bring about sustainable changes in dietary habits (16). ESD aims to develop sustainability competence, enabling learners to think and act sustainably. The goal is to make students aware that their actions impact the world and, accordingly, teach them to make responsible choices (17). As part of the 2030 Agenda for Sustainable Development, the United Nations drafted 17 Sustainable Development Goals (SDGs) in 2015. SDGs encompass areas in which sustainable development should be strengthened, such as sustainable food production and consumption (SDG 12). Understanding the production and consumption of livestock products, such as meat or milk, is an important part of sustainability competence that learners should develop through ESD. Therefore, teaching the topic “Sustainable Nutrition” within ESD offers a great potential for achieving the SDGs (17).

To develop educational implications for teaching specific topics of sustainable development within ESD, an exploration of student conceptions is necessary. There is only a small amount of research on students' conceptions of the production of livestock products, including conceptions of the production of cow's milk. Therefore, this research examines sixth- and tenth-grade students' conceptions of milk production, focusing on students' conceptions of dairy farming, the milking process, milking techniques, and milk production.

### 1.1. Student conceptions, moderate constructivism, and conceptual change

In this study, the basis for studying student conceptions is the theory of moderate constructivism (18, 19). According to this theory, knowledge acquisition is actively constructed by the learner and is based on their pre-existing conceptions (18–20). Students already have simple conceptions about most subjects. These conceptions can be deeply embedded and affect the learning process in class. Therefore, students' conceptions are highly relevant for the didactic and methodological preparation of lessons in science education (19). On the one hand, students' conceptions can be obstacles to learning if they do not correspond to the scientific conceptions. On the other hand, they can form the basis of the learning process and thus promote understanding (18).

Changes in student conceptions can be explained using the conceptual change theory, which is concerned with the conditions under which simple conceptions can change into scientifically justified conceptions (21–24). However, since student conceptions are not simply replaced by scientific conceptions, as “change” implies, Krüger (23) uses the term conceptual reconstruction, which clarifies that conceptions can only be reconstructed, not replaced. According to Posner et al. (24), four conditions must be met for reconstruction of pre-instructional conceptions: (1) the student must be dissatisfied with their existing performance; the new conception must be (2) logical, understandable, and (3) plausible; and (4) the new concept should be fruitful (i.e., applicable to other areas) (19, 23).

For the development of school lessons that build on existing student conceptions, the Model of Educational Reconstruction (MER) is of great use. This model is based on, among other things, the theory of moderate constructivism and the theory of conceptual change (25, 26). The MER is used to design and evaluate an effective learning environment in the classroom by mutually relating existing student conceptions and scientifically justified conceptions. The model is thus composed of three mutually influencing components: (1) the technical clarification of the specific science content, (2) the elicitation of student conceptions regarding the specific science content, and (3) the design and evaluation of learning environments. Under the MER, a balance is struck between knowledge transfer and pedagogical aspects. This enables students to develop appropriate conceptions within the classroom setting (25–27). In this study, we focused on the second emphasis of the MER.

After a brief technical clarification of the topics of dairy farming, the milking process, milking techniques, and lactation, we present the collected student conceptions about milk production and discuss them to formulate practical educational implications. To optimize ESD with the goal of acquiring relevant knowledge, skills, and competencies to achieve the SDGs, the aims of our study are to investigate students' conceptions about the production of an agricultural product (cow's milk) and to compare the conceptions of students of different grade levels/ages as an indicator of their learning gains (SDG 4) (16).

### 1.2. Clarification of science content—Dairy farming in Germany

The stock size of most dairy farms in Germany is between 20 and 49 animals, while the second most common stock size is 100 or more animals (12). An average of 70 cows are kept on a German dairy farm, with the number of dairy cows per farm ranging from 10 to 1,000 (7, 12). Forty percent of dairy cows in Germany have regular pasturing. However, there are regional differences: In eastern Germany and Bavaria, less than 20% of cows have pasturing, while in Lower Saxony, North Rhine-Westphalia, and Schleswig-Holstein, most cows are kept on pasture in the summer (6, 10, 12, 28). The number of dairy farms varies significantly by state. Almost 50% of all German dairy cows are kept in Lower Saxony and Bavaria, with Bavaria having the highest number of dairy farms in Germany (7, 29). A large proportion of milk is produced at sites with a high proportion of grassland, as the grass serves as a feed base for the dairy cows. In terms of volume, the states with the most milk production are Lower Saxony, North Rhine-Westphalia, Schleswig-Holstein, and Bavaria (7, 12).

Regarding the husbandry practices of dairy cows, 83% of dairy cows in Germany are kept in cubicle housings (30). In a cubicle housing system, the cows move freely in the herd and go independently to the milking parlor, the feeding and watering place, and their lying area *via* walking areas. The design of the lying sites must allow for any lying shape of the cow and the animals must be able to stand up and lie down without hindrance. In cubicle housings, slatted floors with rubber mats are usually installed to ensure dryness and sure-footedness for the animals. There is a distinction between cubicle housings with a free lying area and box cubicle housings, in which the individual lying areas are separated with partitions. In dairy farming, cubicle housing has proven its worth, as it requires little bedding, the animals have a protected place to lie down, and the farmer's working time requirement is low (31).

Another husbandry practice is tethered housing, in which the animals are kept exclusively in a confined area where all animal-related activities such as milking, feeding, and manure removal are carried out. In this case, the animals are secured by the neck with a tether. However, the tether must not interfere with the cows' standing up, lying down, or eating. Manure removal is carried out using either the flow manure method, in which the lying surface passes into a grating through which the manure can fall directly into a drifting manure channel and, from there, is directed into a storage container, or solid manure preparation, in which the lying area ends at the manure ditch, into which the manure falls. The manure pit must be regularly mucked out, which can be done by a machine. Due to the animals' lack of freedom of movement, species-appropriate husbandry is only possible to a limited extent in tethered housing (31). In 2020, only 10% of the enclosures for dairy cows in Germany were still in tethered housing. Since tethered housing is no longer being built, it can be assumed that the number of enclosures in tethered housings has decreased further in the course of agricultural structural change (28, 30, 31).

Two other relevant housing types are the calving pen and the calf pen. To ensure hygiene and freedom of movement during birth, a calf should be born in a calving pen. It also serves to build a mother–child relationship. The calf should be transferred to a separate calf pen 24 h after birth (31). The individual housing is justified by a lower risk of infection to the calves. It also allows intensive monitoring of health and feed intake (31, 32).

The formation of milk (lactation) is the most characteristic feature of mammals. The center of lactation is the udder, which consists of four teats; the milk-forming glandular body; a cavity system of teat ducts; milk cisterns; and blood, nerve, and lymphatic systems. With sexual maturity, various hormones cause the development of glandular tissue in the udder. After the first fertilization, pregnancy leads to an increased hormone concentration, which results in the development of milk-forming secretory vesicles (alveoli). Shortly before calving, a change in hormone levels causes the onset of lactation. Capillary forces hold most of the milk in the alveoli and milk ducts, with only a tenth flowing into the cisterns (31).

Cow's milk consists mainly of water, proteins, fats, and carbohydrates in the form of milk sugar (lactose). Nutrients ingested in food are filtered from the blood by the alveoli. The amino acids filtered from the blood are ultimately used to form the milk proteins. The synthesis of milk fat also takes place in the alveoli. To produce 1 liter of milk, 400 to 600 liters of blood must pass through the udder (31).

A dairy cow becomes pregnant for the first time at approximately 18 months of age. Fertilization occurs *via* artificial insemination in 90% of dairy cows in Europe. On German organic farms, 80–90% of dairy cows are artificially inseminated. Natural sprouting is mainly used by extensively managed farms or organic farms, or in the case of fertility problems (33, 34). A dairy cow is pregnant for approximately 9 months and is milked for about 10 months after birth. The cow is inseminated again shortly after birth to avoid long dry stall periods. The milk yield of a cow depends on her age, since the milk-forming cells (alveoli) multiply with each pregnancy (31, 35). Furthermore, milk yield depends on the dairy breed (36). For example, the dairy breed Holstein (*Bos taurus taurus)* has a higher milk yield (9,224 kg milk/year) than the dairy breed Jersey (6,428 kg milk/year) (36). Physical conditions like pain, metabolic diseases, stress, separation of mother and young animal, or inflammation can have a negative effect on milk yield (31). Today, a dairy cow in Germany has an average of two to three calves and is ultimately slaughtered after about 4.5 years (10, 28).

To prepare for milking, the teats are actively stimulated by pre-milking them. This eventually causes the milk to shoot from the alveoli into the teats. The greater the time interval between milking, the greater the pressure in the udder, which can inhibit milk production. Thus, milk yield can be increased through more frequent milking (31). Dairy cows are milked 2 to 3 times a day, depending on milk yield (28). The milking process takes between 5 and 8 min. In addition to machine milking, a few farms still milk by hand. However, this is usually limited to pre-milking or performed the udder needs to be completely emptied in cases of udder disease (31).

Semi-automated milking plants or automatic milking systems (AMS) are used for milking. If the milker is relieved of individual work steps by technical systems, a milking plant is considered to be partially automated. Milking plants are considered AMS or milking robots if all steps of the milking process occur without a milker's intervention. AMS do not require fixed milking times, as the cows visit the milking plant independently. The animals are lured in with feed to maintain regular and timely milking. AMSs can accommodate from 20 to 48 animals. They clean themselves automatically between milkings and are ready for use 24 h a day, allowing for more than two milkings per day (31).

### 1.3. Current state of research—Student conceptions of “milk production”

As part of her dissertation, Hamann (37) investigated fourth graders' conceptions of agriculture in the context of ESD. In the study by Hamann (37), the cow was the most familiar of all farm animals. Almost all children knew that a cow provides milk. Furthermore, the elementary school students were able to describe in detail the housing of pigs, chickens, and cows. The idea that cows are largely kept on pasture and can switch between barn and pasture prevailed. The students linked this conception to the wellbeing of the animals. Most students assumed that the animals would be kept indoors only at night, during bad weather, or in case of illness. Overall, correlations were found between the students' primary and secondary experiences with farms and their conceptions. It also became apparent that real-life encounters with agriculture shaped the children's conceptions more than any other influencing factors (37).

In an interview study, Folsche and Fiebelkorn (38) investigated the conceptions of six elementary school students regarding the keeping of fattening pigs and dairy cows. Three of the students grew up on a conventional fattening pig farm (farm students). The survey revealed different conceptions of farms, from romanticized to elaborated. Students who did not grow up on a farm (urban students) associated keeping fattening pigs and dairy cows with small stock sizes and access to an outdoor run. In contrast, the farm students had realistic conceptions about stock sizes, barns with slatted floors, and technical equipment (e.g., automatic feeding). Primary experiences were shown to have a significant influence on the students' conceptions (38).

The research accompanying the Youth Report Nature 2010 by Brämer (39) provides an overview of the state of research on children's conceptions about agriculture. In a ranking scale of farm animals, the cow was the most frequently mentioned. Likewise, milking and the product associated with it (milk) were mentioned particularly often in relation to farm animal handling. It was found that children have romanticized conceptions of farms without technical equipment. Furthermore, students' knowledge about livestock farming came mainly from parents and media, while curricular school content had only a small effect on knowledge acquisition (39).

Schütte and Busch (40) investigated the implementation of the topic of agriculture in school lessons at high schools and grammar schools in Lower Saxony. According to this study, approximately 90% of the teachers surveyed stated that agricultural topics are included in the school's internal curriculum, but there were differences in the regularity of the implementation of lesson series with an agricultural focus: Only just under half of teachers taught agricultural topics on a regular basis. This topic was taught most frequently in grades five and six, followed by grades nine and ten. Sixty percent of the teachers surveyed went on a field trip with their classes, with farms being the most frequent destination (40).

### 1.4. Aims of the study

As indicated by the current state of research, studies on elementary school students' conceptions of agriculture (37) and the keeping of fattening pigs and dairy cows have already been published (38). However, to our knowledge, no study has yet examined students' conceptions of milk production. Thus, the following research questions were formulated:

- Research Question 1: What conceptions do students have of keeping dairy cows?
- Research Question 2: What conceptions do students have of the milking process and the technology used?
- Research Question 3: What conceptions do students have of milk production in dairy cows?

The focus of this paper is to compare the conceptions of sixth- and tenth-grade students. This comparison can be used to determine learning gains between sixth and tenth graders.

## 2. Materials and methods

### 2.1. Sample

The sample included four students each from the sixth (*n* = 4; *M*_*Age*_ = 12 years; *SD*_*Age*_ = 0.7 years; 50% female) and tenth grades (*n* = 4; *M*_*Age*_ = 16 years; *SD*_*Age*_ = 0 years; 50% female) from one grammar school in the city of Osnabrück (Lower Saxony, Northwest Germany). To ensure the anonymity of the students, a pseudonymization was performed. None of the students lived on a farm at the time of the interviews. A more detailed description of the sample and their primary and secondary experiences with dairy cow farms can be found in Tables 1, 2.

**Table 1:** Overview of sixth-grade students.

**Name, age, gender**	**Primary experiences**	**Secondary experiences^1^**
Anna, 13 years, female	Relatives' small farm in their home country	High school: Cow stomachs
Johannes, 11 years, male	Class trip to farm in elementary school	High school: Ruminants (one lesson)
Mark, 12 years, male	Regular vacation on Austrian alpine pasture, can milk cows and make dairy products there yourself	High school: Ruminant stomachs, cloven-hoofed animals, farm, sustainability; watches knowledge shows at home
Marie, 12 years, female	Grandmother's dairy farm in her home country; field trip to dairy farm in elementary school: tour of the milking equipment and independent milking	Elementary school: Stomachs of the cow (uncertain)

^1^Self-reported secondary experiences. Some students had limited recollection of secondary experiences with (dairy cow) farms, so these statements were marked with “uncertain”.

**Table 2:** Overview of tenth-grade students.

**Name, age, gender**	**Primary experiences**	**Secondary experiences^1^**
Eva, 16 years, female	Father grew up on a farm with few cows; vacationed on a farm with cows in South Tyrol and was allowed to milk cows there	Elementary school (uncertain)
Kristina, 16 years, female	Farm vacation as a child	Elementary school (uncertain)
Michael, 16 years, male	Was sometimes in a farm shop/farm restaurant, farm kept fattening bulls	Biology class (uncertain)
Jürgen, 16 years, male	Was allowed to try a lot while working with dairy cows, pigs, and chickens on vacation	Elementary school (uncertain)

^1^Self-reported secondary experiences. Some students had limited recollection of secondary experiences with (dairy cow) farms, so these statements were marked with “uncertain”.

The sample is a convenience sample whose size was determined by the availability of the schools in the city of Osnabrück (41). For recruitment, a general call for participation in the study was launched at Osnabrück city schools. One school responded to the general call for participation and selected four students each from the sixth and the tenth grades in a self-selection process. All students volunteered for the interview and were selected by a teacher using a lottery. The students were not known to the interviewers before the start of the study. Written consent was obtained from both the school administration and the participants' legal guardians before the study was conducted. The students gave verbal consent for the interviews in advance.

### 2.2. Data collection

Data collection took place in June 2018. The interviews with the sixth graders were conducted by the fourth author, while the interviews with the tenth graders were conducted by the third author. Interviews were recorded with two digital recording devices (Olympus LS-P1). To optimize the interview questions and the interview procedure, two interviews were held in advance with a 12-year-old sixth grader and a 15-year-old tenth grader in May 2018. To elicit student conceptions of milk production, students completed a schematic of a cow and an udder during the interview (see Section 3.3.2., **Figure 9**). In addition, after the interview recording, a brief questionnaire was conducted with each student to ask about sociodemographic information, primary and secondary experiences with dairy farms, and dairy product consumption. The duration of the interviews ranged from 15 to 25 min (*M*_*Duration*_ = 18.4 min; *SD*_*Duration*_ = 3.1 min) for sixth graders and from 29 to 48 min (*M*_*Duration*_ = 38 min*; SD*_*Duration*_ = 8.5 min) for tenth graders. Interviews were conducted in German and translated into English for the purposes of this study. The study was conducted in accordance with national and institutional guidelines, the Declaration of Helsinki, and guidelines from the German Research Foundation and the American Psychological Association. Participants' anonymity was assured, and participation was voluntary (42, 43). All participants had the opportunity to decline to participate in the study at any time and without any consequences. All materials used to conduct the interviews were provided to the students' guardians prior to conducting the interviews, and all questions were clarified during the interview. The content of the interview guide was not shared with the students. Our research will not affect the rights and welfare of our participants, and no sensitive personal information was assessed. Thus, an ethics approval was not required by institutional and national guidelines (42).

### 2.3. Interview procedure and study design

**Figure 1:**
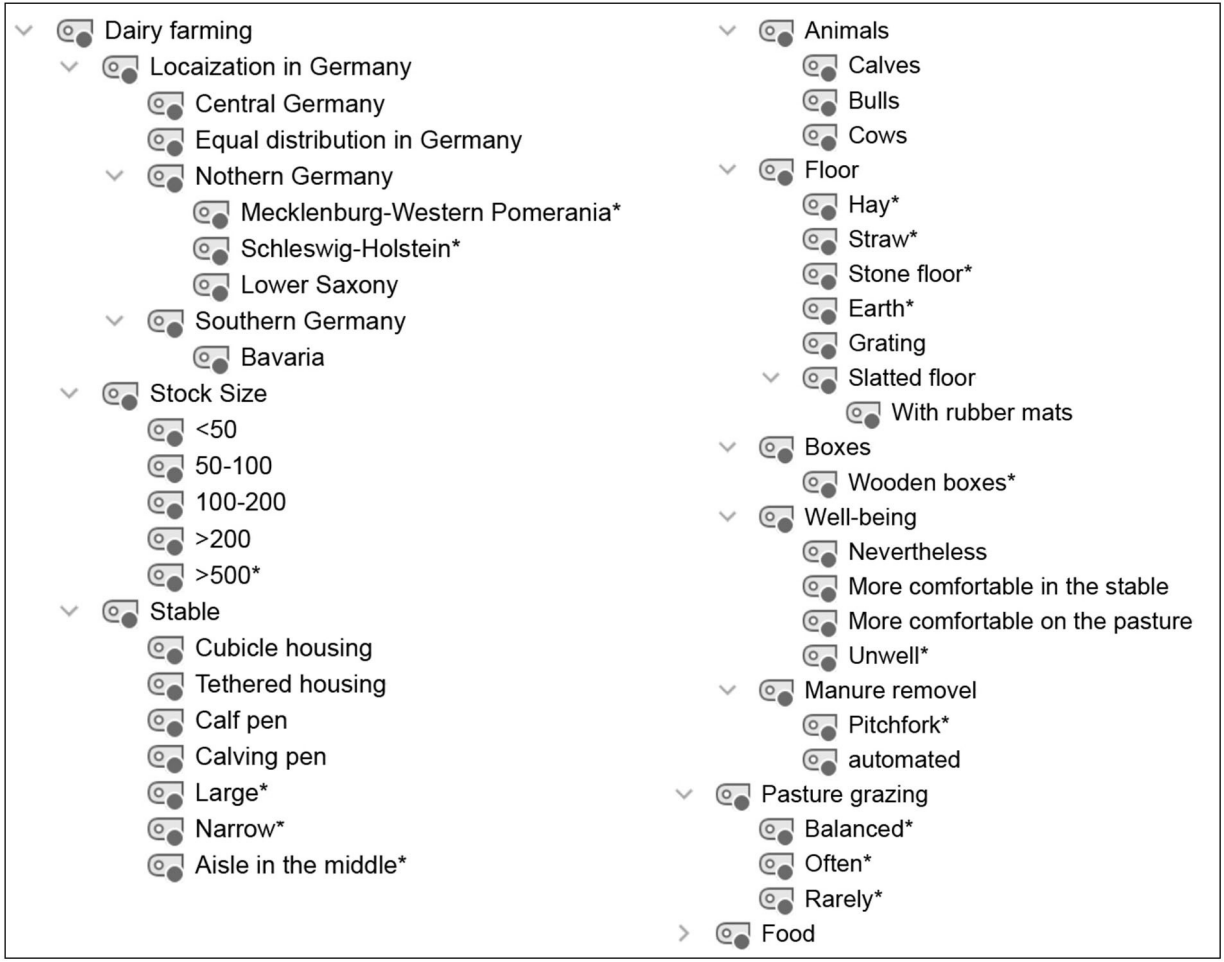
Category system for analyzing student conceptions of dairy farming. ^*^Inductively coded categories.

**Figure 2:**
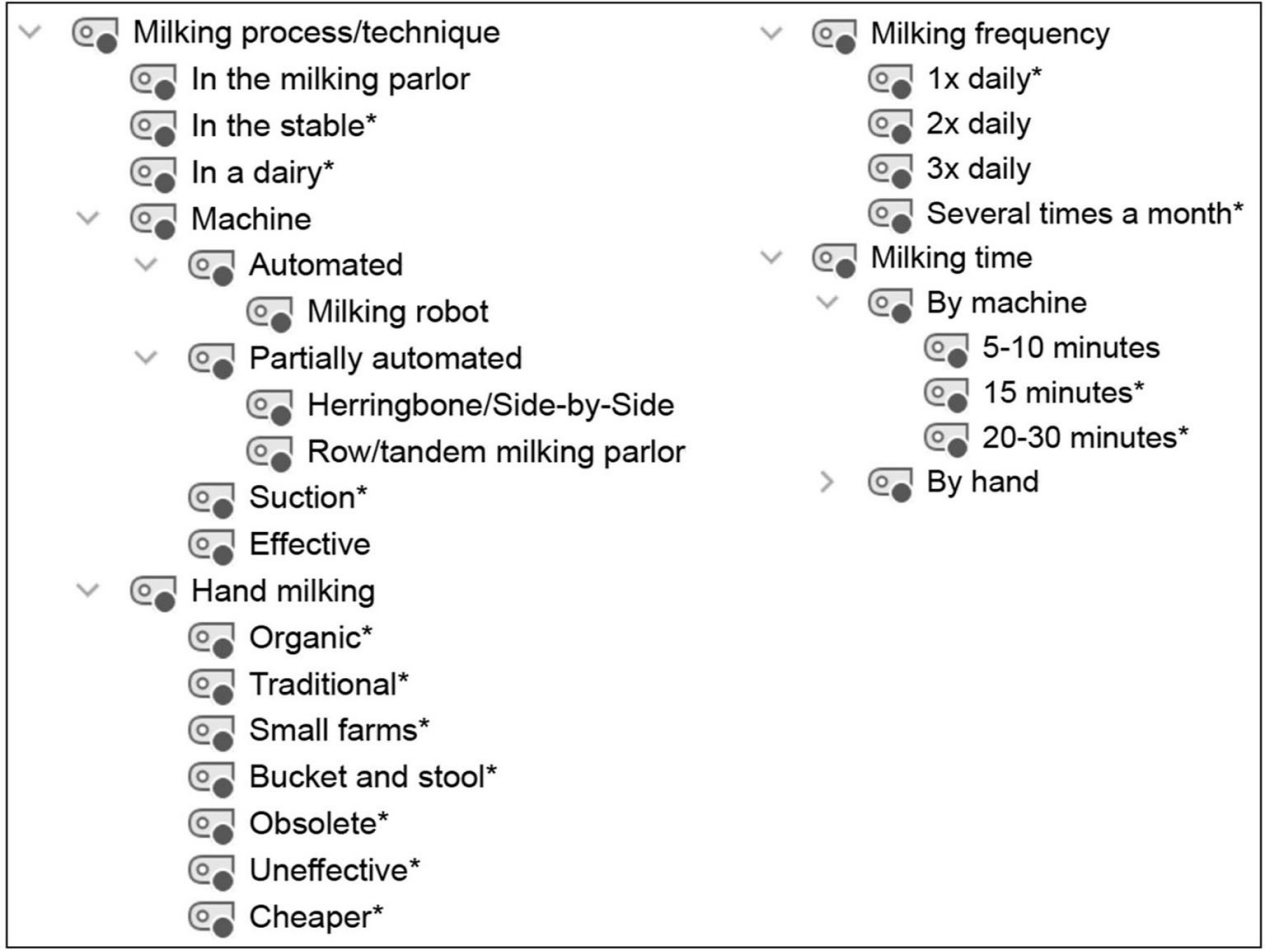
Category system for analyzing student conceptions of the milking process/technique. *Inductively coded categories.

**Figure 3:**
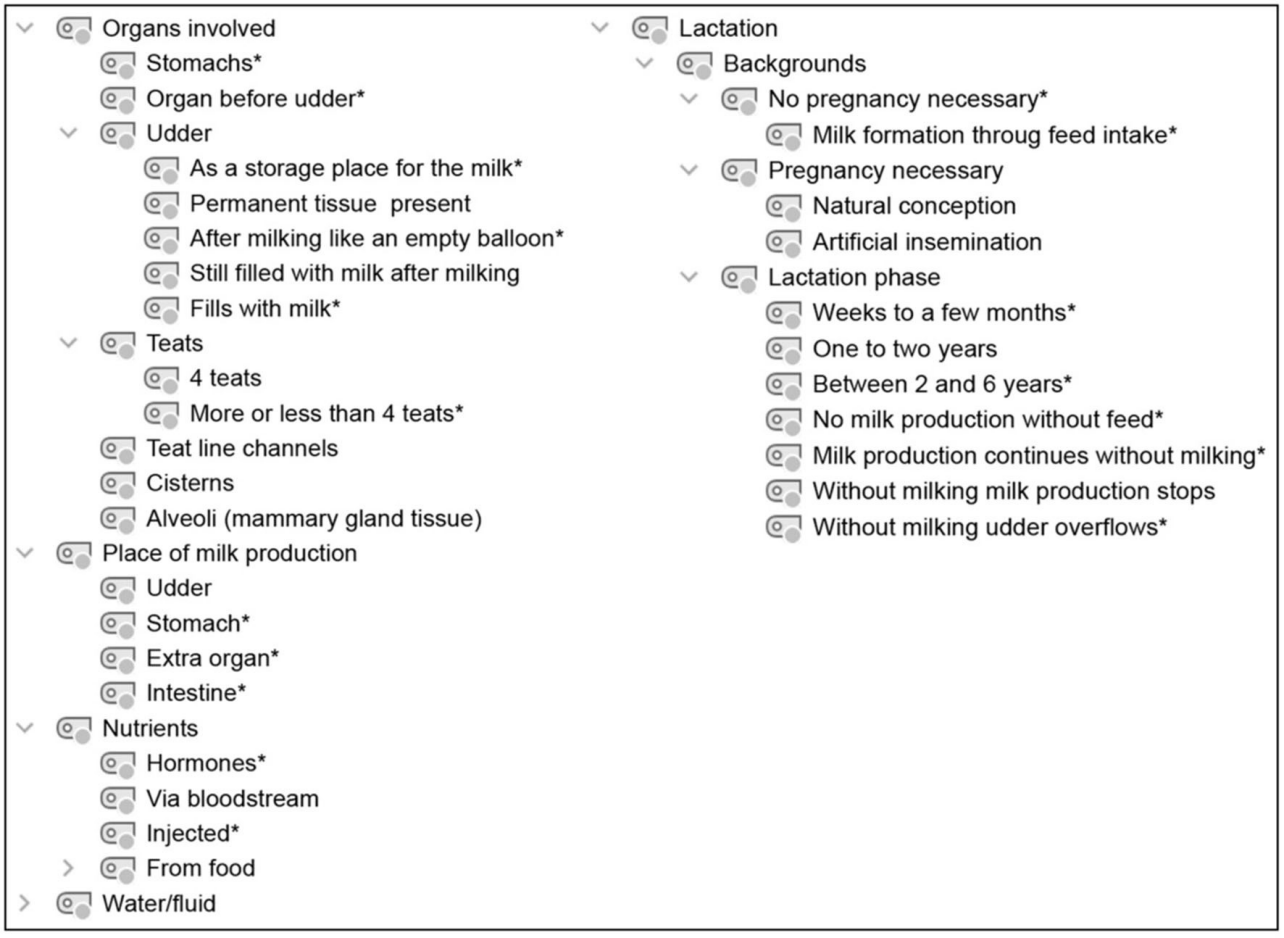
Category system for analyzing student conceptions of lactation. *Inductively coded categories.

To collect the students' conceptions, an interview study was conducted. A semi-structured interview guide was used to ensure comparability of data, and a multi-method interview approach combining drawing and verbal questioning was used (37, 38, 44). In general, the interviews were divided into three parts: (1) an introduction to the study, (2) the main part in which questions about students' conceptions of milk production were asked, and (3) a closing, which included a short questionnaire. The main part of the interview consisted of three phases relevant to the three research questions: (1) student conceptions about dairy farming, (2) student conceptions about the daily routine of a farmer on a typical dairy farm, and (3) student conceptions about milk production in a dairy cow. In the first phase, the students' conceptions of dairy farming were surveyed. For this purpose, the students were asked about their conceptions of dairy farms in Germany, the structure of a typical dairy farm, and the keeping of dairy cows. The focus was on the localization of dairy farms in Germany, stock sizes, the design of cow housing, and pasture grazing (Figure 1). In the second phase, students' conceptions of the daily routine of a farmer on a typical dairy farm were surveyed. The focus here was on asking the students about the milking process and milking techniques (Figure 2). In the third phase, the students' conceptions about the milk production of a dairy cow were surveyed. This involved first ascertaining the students' conceptions of why a cow produces milk and then asking how milk is produced in the cow (Figure 3). The latter was to be depicted by the students in drawings during the interview. For this purpose, students completed a schematic of a cow and an udder in which they could draw their conceptions about milk formation (see Section 3.3.2., **Figure 9**). This was followed by a review and discussion of the drawing. The statements of the students were further deepened with the help of a questionnaire covering different topics in dairy farming, milking, and milk formation.

### 2.4. Data processing and analysis

Audio recordings of the interviews were transcribed using the transcription program f4transcript according to the guidelines of Dresing and Pehl (45). The transcripts were analyzed in MAXQDA (46) using qualitative content analysis according to Kuckartz (47). Based on the research questions and three phases of the interview, a deductive category system was created according to the clarification of the technical concepts with the superordinate categories of (1) dairy farming (Figure 1), (2) milking process/techniques (Figure 2), and (3) lactation (Figure 3). In addition, inductive categories were added to the deductively created category system of technical conceptions during the analysis process.

For research economy, coding of all statements by a second person and using intercoder agreement as a consistency check were not performed. However, it can be assumed that coding by a second person would reveal only minor differences, since the deductive codes of the coding family tree correspond to a large extent to the questions on the interview guide and the questionnaire.

## 3. Results

**Figure 4:**
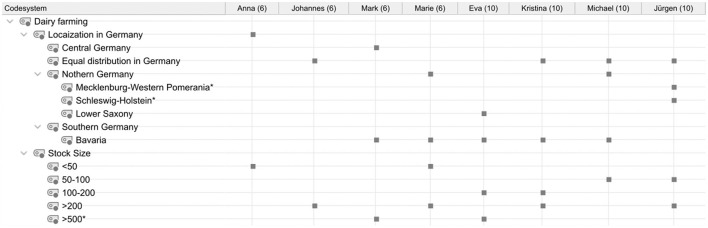
Overview of the students' statements on the localization of dairy farms in Germany and their stock sizes. *Inductively coded categories. The marks by the categories indicate that they were named by the student.

**Figure 5:**
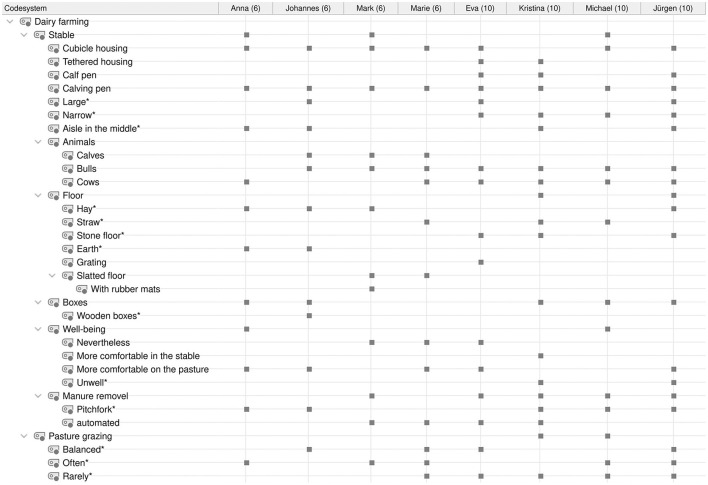
Overview of the students' statements on stable and pasture husbandry. ^*^Inductively coded categories. The marks by the categories indicate that they were named by the student.

**Figure 6:**
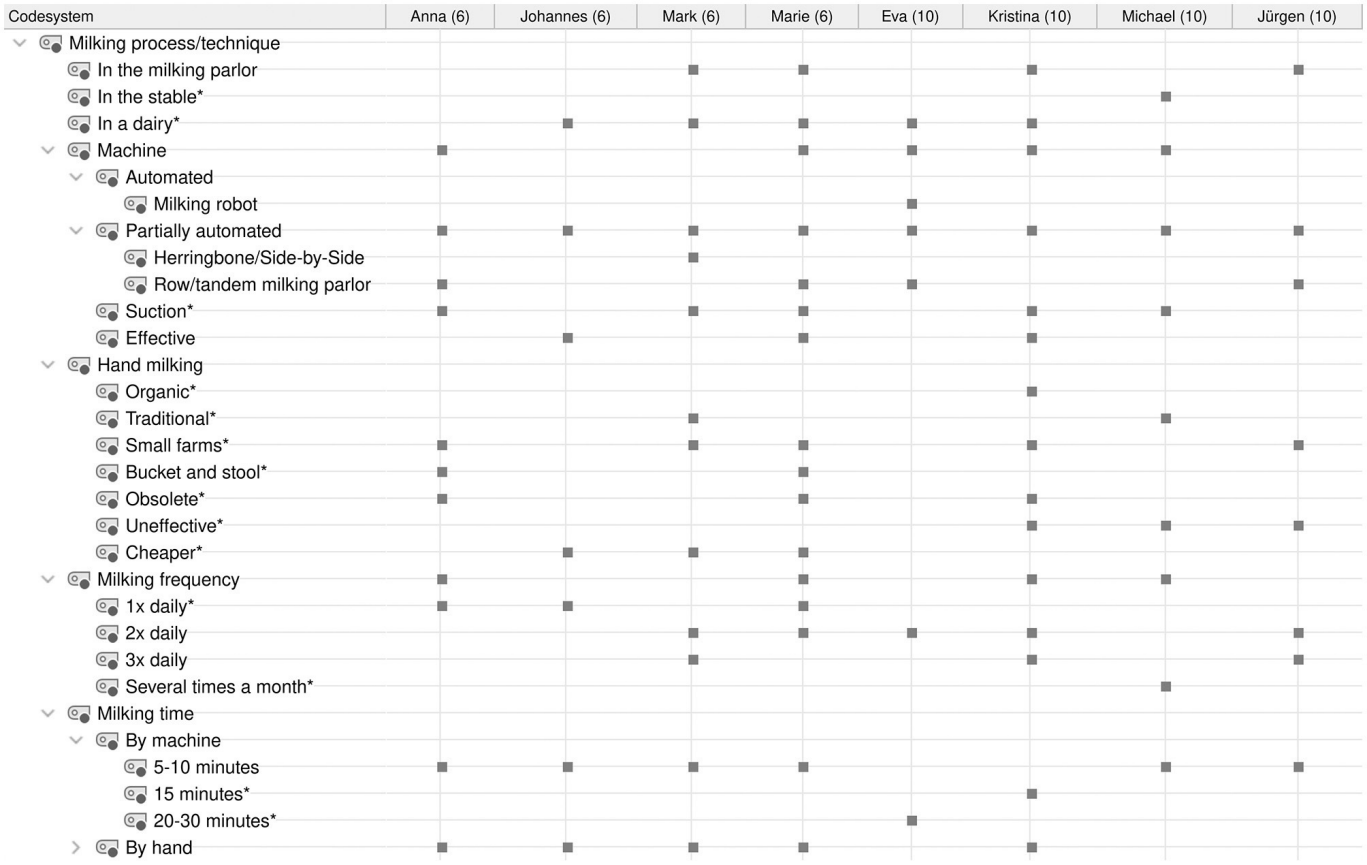
Overview of the students' statements on the milking process and milking techniques. ^*^Inductively coded categories. The marks by the categories indicate that they were named by the student.

**Figure 7:**
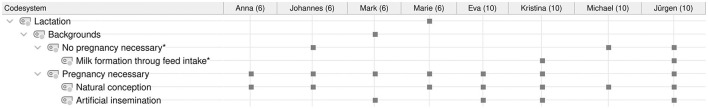
Overview of the students' statements on the reasons for milk production. ^*^Inductively coded categories. The marks by the categories indicate that they were named by the student.

**Figure 8:**
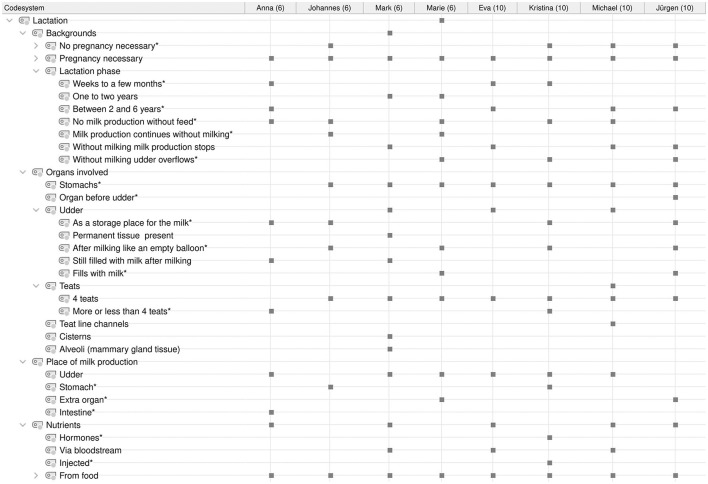
Overview of the students' statements on the process of milk formation in a cow. ^*^Inductively coded categories. The marks by the categories indicate that they were named by the student.

Student conceptions were sorted and summarized based on the research questions and using the category systems (Figures 4–**8**). Students' statements were assigned to the corresponding categories, with an indication of their grade level after the pseudonym in round brackets in the category system.

### 3.1. Students' conceptions of keeping dairy cows

#### 3.1.1. Localization and stock size of dairy farms in Germany

The statements of all students on the localization of dairy farms in Germany as well as their typical stock size are shown in Figure 4.

Half of the students assumed an equal distribution of dairy farms across Germany. Some of them elaborated on their statements regarding the localization of dairy farms in Germany. Most of the students assumed a relatively high localization of dairy farms in the federal state of Bavaria. Marie (6) and Michael (10) also indicated that dairy farms are localized in northern Germany, while Mark (6) also mentioned central Germany and Jürgen (10) added Schleswig-Holstein and Mecklenburg-Western Pomerania. Mark (6) justified his conception with the fact that dairy farms are located near many meadows and that “better grass” grows on mountains. Eva (10) was the only student who explicitly stated that many dairy farms are located in Lower Saxony.

The students estimated that stock sizes on a typical dairy farm ranged from 20 to 30 animals on small farms to over 500 animals on large farms. Mark's (6) statement is particularly striking, as he assumed stock sizes of 1,000 to 1,200 animals.

#### 3.1.2. Design of the cowsheds and pasture grazing

The statements of all students concerning stalls and pasture grazing of dairy cows are shown in Figure 5.

Except for Kristina (10), all students mentioned characteristics of cubicle housing. Johannes (6) and Anna (6) described that the cows can move freely in the stable. Anna (6) also emphasized that the cows have a lot of space to get to the pasture through a passageway. In Marie's (6) conception, the cows have the opportunity to walk to the feeding area. Anna (6) and Johannes (6), as well as Kristina (10), Michael (10), and Jürgen (10), described the animals being kept in boxes. Eva (10) and Kristina (10) mentioned characteristics of tethered housing. For example, Eva (10) described that “exactly one cow fits between two partitions,” while Kristina (10) assumed that the animals cannot turn around or lie down in the barn. In their statements, both emphasized that the cows in the barn are kept in a very confined space.

A common conception of all students was a separate barn for pregnant cows to which they are brought shortly before birth at the latest and where the calves are born. Anna (6), Marie (6), Mark (6), Johannes (6), and Michael (10) also assumed that the cow and her calf would stay together in this separate barn for a certain time after birth. Eva (10), Kristina (10), and Jürgen (10), on the other hand, described that the cow and calf are separated shortly after birth and that the calves are subsequently kept in a calf pen. All students except Anna (6) assumed that at least one bull is kept on a dairy farm in addition to cows.

Regarding conceptions of the barn's layout, few differences were evident between the sixth and tenth graders, with most students describing cubicle housing. Similarly, all students imagined a calving pen. However, differences can be observed with regard to the cohabitation of cow and calf after birth: All sixth graders, as well as Michael (10), assumed that the cow and the calf would stay together. In contrast, Eva (10), Kristina (10), and Jürgen (10) assumed that they would be separated soon after birth.

The individual student conceptions of the stable floor varied. Anna (6) and Johannes (6) described a floor made of soil, while Kristina (10) and Jürgen (10) imagined a stone floor. For Michael (10), the floor is covered with straw, whereas Eva (10) assumed that the floor would have a grid for the drainage of feces and urine. Marie (6) and Mark (6) imagined a slatted floor. Mark (6) also described rubber mats in the stall areas.

Most of the students thought that cows feel more comfortable in the pasture than in the barn. However, Mark (6), Marie (6), and Eva (10) also believed that cows can also feel comfortable in the barn. For Kristina (10), pasture grazing is rarely if ever an option, so she believes that the animals feel uncomfortable overall, but “put up with being kept indoors.” With the exception of Kristina's (10) conceptions, no tremendous year-specific differences could be found concerning conceptions of animal welfare.

The students assumed that dairy farming takes place predominantly on pasture. With the exception of Kristina (10), all students imagined a passage from the barn to the pasture. For Jürgen (10), pasture grazing is limited to organic farming. Accordingly, he had more differentiated conceptions than the sixth graders. In contrast, Kristina (10) and Eva (10) believed that the cows rarely if ever graze outside. Their conceptions thus differ significantly from those of the rest of the participants.

### 3.2. Students' conceptions of the milking process and milking technique

The statements of all students regarding the milking process and milking techniques are shown in Figure 6.

All students imagined a mechanical milking process utilizing partially automated milking plants. Eva (10) was the only student who additionally assumed that a fully automatic milking robot takes over the work of the dairy farmer. In this context, five students associated the verb “suck” with the mechanical milking process. Moreover, the students stated that the mechanical milking process is more effective than milking by hand. Hand milking was considered somewhat outdated or traditional by the students. According to Anna (6), Mark (6), Marie (6), Kristina (10), and Jürgen (10), hand milking is used only by small farms with a few cows. Johannes (6), Mark (6), and Marie (6) suspected that farms practice milking by hand for cost-related reasons, as it seemed to be cheaper than buying a milking machine. In the conceptions of Anna (6) and Marie (6), milking by hand is classically practiced with a bucket and stool.

Regarding milking frequency, most of the students imagined that a cow is milked one to three times a day. What stands out is the statement by Michael (10), who assumed that a cow without a calf is milked once a month and a pregnant cow or one that has already calved is milked three to four times a month.

Regarding the duration of milking with a milking machine, the students estimated that it generally takes 5 to 10 min per cow. However, Kristina (10) and Eva (10) assumed a milking time of 15 to 30 min. Apart from Michael's (10) conceptions of milking frequency, no significant year-specific differences could be found regarding overall conceptions about the milking process and milking technique.

### 3.3. Students' conceptions of milk production in dairy cows

#### 3.3.1. Reasons for milk production

The statements of all students on the reasons for milk production are shown in Figure 7.

Regarding the reasons for milk production, Anna (6), Mark (6), Marie (6), and Eva (10) stated that a cow typically produces milk for her calf and therefore has to calve once. Johannes (6), Kristina (10), and Jürgen (10) were not aware of this at first. After a hint from the interviewer, however, they also independently concluded that a cow must have once been pregnant to produce milk. Michael (10), on the other hand, believed that cows already produce milk “passively” before the first calving and that production is enhanced by a pregnancy and a calving. This is what Michael (10) called a “passive side effect of being a cow mother.” He added that the milk is drunk by the calf and that milk is “taken away” from it by milking.

All students except Mark (6) basically imagined natural insemination by a bull. Eva (10), Kristina (10), and Jürgen (10) also considered artificial insemination, which Eva (10) associated with large farms, in particular. Jürgen (10) understood artificial insemination to mean that “genetic materials” are inserted into the cow or that they are ingested through the feed.

Overall, the tenth graders had more differentiated conceptions about why a cow produces milk. They could imagine artificial insemination in addition to natural insemination, while the sixth graders anticipated only natural conception. Mark (6) was the only respondent to rule out natural insemination. He had precise conceptions about the process of artificial insemination, explicitly naming a technician for artificial insemination methods.

#### 3.3.2. Process of milk production

**Figure 9:**
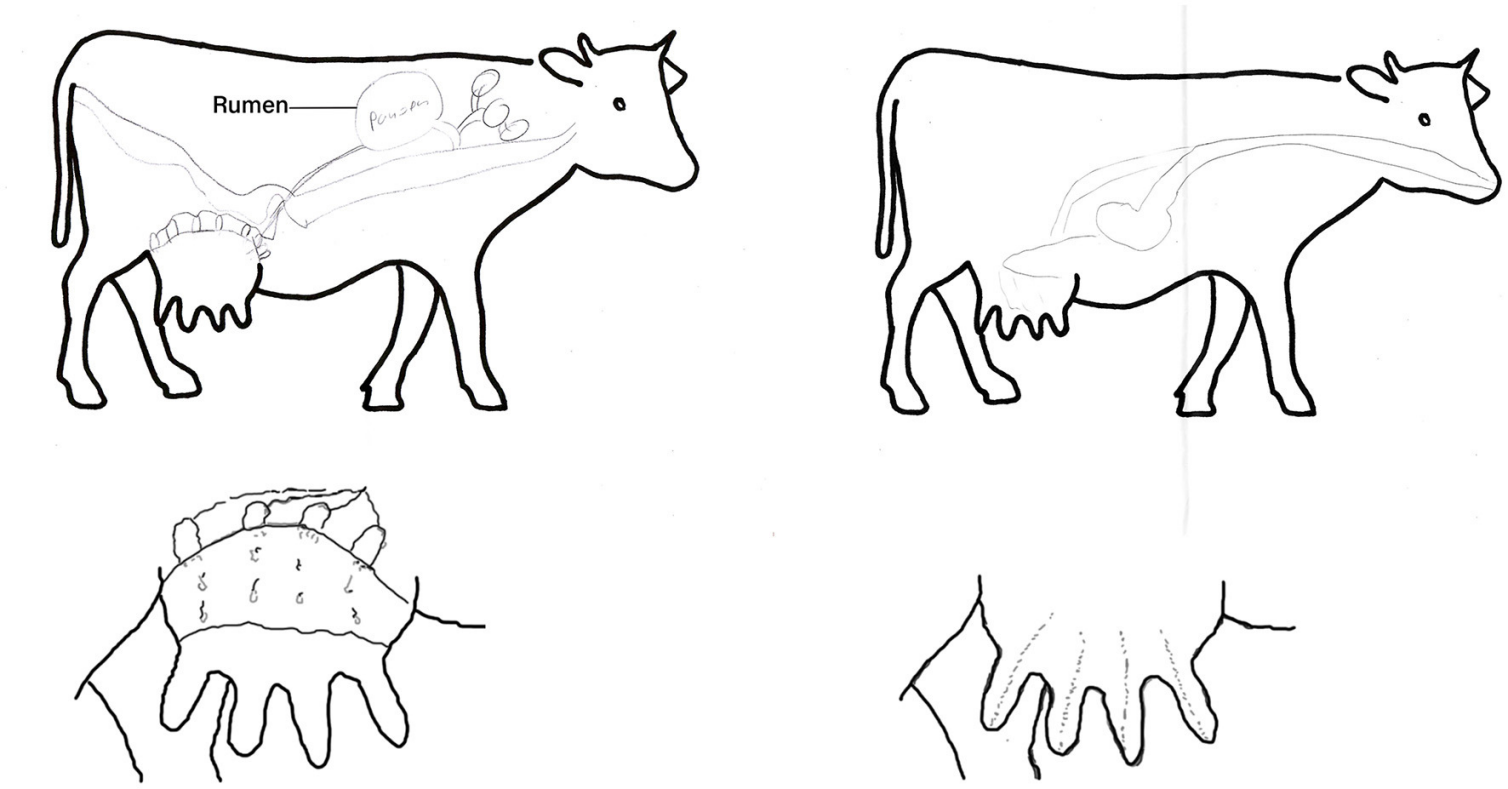
Drawings by Mark [6th grade, 12 years; **(left)**] and Michael [10th grade, 16 years; **(right)**] of milk production in the body and udder of a cow.

The lactation process was described and outlined very differently by different students. The expressions of all students on the process of milk formation are shown in Figure 8. Figure 9 shows two exemplary drawings of students' conceptions of milk production in a cow's udder by Mark (6) and Michael (10).

All students except Anna (6) named the cow's stomachs, which are responsible for nutrient utilization, and the udder as organs involved in milk production. Marie (6) and Jürgen (10) imagined that milk production itself occurs in a separate organ in front of the udder, while Johannes (6) and Kristina (10) stated that milk production occurs in one of the stomachs. For Anna (6), milk production takes place in the intestine. Mark (6) was the only student to describe the mammary gland tissue and the cisterns in the udder. He described that the udder is mainly responsible for milk production. Johannes (6), Marie (6), Kristina (10), and Jürgen (10) imagined that the udder is like an empty balloon after milking. Only Mark (6) believed that the udder is permanently made of tissue and that some milk remains in it even after milking.

All students except Anna (6) described an udder with four teats. Anna (6) described an udder with six or seven teats. Kristina (10) also considered an udder with more than four teats, although she described it as unusual.

There was a predominant consensus that the nutrients in milk are absorbed from food. Anna (6), Johannes (6), Marie (6), Kristina (10), and Michael (10) were also convinced that a cow must ingest food as a prerequisite for milk production. Furthermore, Mark (6), Eva (10), and Jürgen (10) stated that milk yield can be increased by special feed. According to Kristina (10) and Michael (10), the feed significantly affects milk quality.

Overall, it is clear that the students' conceptions of the lactation process vary widely.

## 4. Discussion

### 4.1. What conceptions do students have of keeping dairy cows?

#### 4.1.1. Localization and stock size of dairy farms in Germany

The results reveal different students' conceptions regarding the localization of dairy farms in Germany. Most of the students imagined that dairy farms are predominantly in Bavaria. In addition, Marie (6) and Michael (10) named northern Germany, while Mark (6) also mentioned central Germany and Jürgen (10) localized dairy farms in Schleswig-Holstein and Mecklenburg-Western Pomerania. This roughly corresponds to the actual distribution of dairy farms in Germany, as the most significant milk production occurs in Bavaria, Lower Saxony, North Rhine-Westphalia, and Schleswig-Holstein (7, 12). Although about half of all German dairy cows are kept in Bavaria and Lower Saxony (7, 12), Lower Saxony was only associated with increased dairy farm establishment by Eva (10). Thus, Bavaria was the federal state primarily associated with dairy farming by the students in this study, even though dairy cows in Lower Saxony graze more frequently in percentage terms than those in Bavaria (7, 12). Moreover, given that the students lived in Lower Saxony, it would be expected that dairy farming in Lower Saxony would be more present in students' conceptions. Thus, the regional correlation between students' hometowns in Lower Saxony and the high density of dairy cattle in northwestern Germany turned out to be weaker than expected. This might be due to the influence of the marketing of cow's milk, which tends to convey an idyllic image of grazing cows on an alpine pasture rather than cows in the plains. The students' conceptions of stock size (from 20 to over 200 animals depending on farm size) are quite close to actual stock sizes; most dairy farms in Germany keep between 20 and 49 animals or over 100 animals (12). These realistic conceptions are consistent with the results of a study on conceptions of stock sizes size of fattening pigs, dairy cows, and laying hens by Folsche and Fiebelkorn (38). On the other hand, Mark (6) expressed the conception of intensive livestock farming, as he could only imagine dairy farms with a stock size of 1,000 to 1,200 animals. These results are in contrast with the findings of Hamann (37), who could not identify any conceptions of industrial farming with stock sizes of several thousand animals among the students surveyed.

#### 4.1.2. Design of the cowsheds and pasture grazing

Regarding housing, almost all students described dairy cattle being kept in cubicle housing, where the animals can move freely. This is in line with common husbandry practice in Germany, as 83% of dairy cows are kept in cubicle housing (12). In the context of cubicle housing, most of the students imagined that the animals can choose between the stall and pasture utilizing a passageway. This is in line with the results reported by Hamann (37) and Folsche and Fiebelkorn (38). The students indicated that the cows can choose between being outdoors and staying indoors at will. This is matched by the students' conceptions of dairy farming as a combination of confinement and pasture grazing. Thus, their conceptions reflect the typical landscape of Northwest Germany of dairy cows on pastures (12, 37, 38). However, since the students hardly associated Lower Saxony or Northwest Germany with dairy farms, this idea can be described as “idyllic.”

Kristina (10) and Eva (10) showed that the keeping of dairy cows can also be associated with less species-appropriate factory farming. In their conceptions, tethering dominates. Notably, however, the number of cows kept in tethered housing has decreased by 62% since 2010 (30). Their negative conceptions could have been influenced by primary experiences with dairy farms, as the quality of (dairy) farm visits influences conceptions of agriculture (37, 48, 49).

All students expressed the realistic view that pregnant dairy cows are kept in a calving pen shortly before calving and give birth to the calf there. In more differentiated responses, Eva (10), Kristina (10), and Jürgen (10) described that the cow and calf are separated promptly after birth and the calves are subsequently kept in a calf pen. These conceptions are in line with real-life practices on dairy farms after calving (31). In contrast, Anna (6), Marie (6), Mark (6), Johannes (6), and Michael (10) had romanticized conceptions about practices on dairy farms after calving, as they imagined that the cow and calf stay together for a longer period after birth.

The students' descriptions of the stable floor varied widely. Marie (6) and Mark (6) imagined a slatted base, which in Mark's (6) description is additionally covered with rubber mats. This image is very realistic, as it corresponds to the typical floor of cubicle housing (31). Also realistic is Eva's conception of a floor with grating to drain feces and urine. This description corresponds to the ground in tethered housing when manure is removed using the flow manure method (31). Notably, Folsche and Fiebelkorn (38) found realistic conceptions of a barn floor only among children who grew up on farms. Accordingly, their results are not in line with the realistic conceptions of Marie (6), Mark (6), and Eva (10), who only gained primary experiences on farms during vacations or when visiting relatives. In interviews with the other students, a floor consisting of earth, a stone floor, and a floor exclusively covered with straw were described. These conceptions are less realistic and can be compared to the research findings of Hamann (37), who described that children anticipated a floor covered with hay or straw.

Regarding animal welfare, Kristina (10) stated that grazing was rarely (if ever) an option, as this might explain why she stated that animals feel unwell overall. This conception can be explained by the findings of Folsche and Fiebelkorn (38). In their study, students stated that domestic animals kept only indoors have less space and are not well due to limited movement. In the current study, the remaining students' conception was that the wellbeing of the animals is higher in the pasture than in the barn. This reflects the view of the majority of the students in this study that dairy farming is predominantly pasture-based. Mark's (6) and Marie's (6) conception of the animals' feeling equally comfortable indoors can be justified by the fact that in their conceptions, the animals are kept in cubicle housing in which the animals do not suffer from lack of space and movement restrictions.

### 4.2. What conceptions do students have of the milking process and the technology used?

All students' conceptions about milking systems corresponded to the methods used in Germany for milking: partially automated milking systems or AMS (31). They described a milking process carried out mechanically and employing partially automated milking plants. Eva (10) was the only student who could also imagine a milking robot. The machine milking process was considered by the students to be more effective than traditional milking by hand. These results contrast with the findings of Brämer (39), who identified a romanticized conception of farms without technical aids in the majority of students surveyed. Therefore, most students have increasingly realistic ideas of industrialized agriculture with automated milking equipment.

Students' conceptions of the duration of machine milking are predominantly consistent with milking durations in partially automated milking systems (31). In addition, most of the students also had realistic conceptions about milking frequency, as dairy cows are milked two to three times per day (28). In contrast, Michael (10) believed that a cow that has never calved is milked once a month, while a pregnant cow or one that has already calved is milked three to four times a month. Michael's example shows a romanticized idea of livestock farming with a small stock size on dairy farms and a very low milking frequency.

### 4.3. What conceptions do students have of milk production in dairy cows?

#### 4.3.1. Reasons for milk production

Anna (6), Mark (6), Marie (6), and Eva (10) immediately associated the reason for a cow's milk production with the fact that a cow typically produces milk for her calf and consequently has to calve at least once. Accordingly, the four students had realistic conceptions about the reasons for lactation (31).

Johannes (6), Kristina (10), and Jürgen (10) could only explain the reason for cow's lactation after a hint from the interviewer. One reason for this could be that the students were aware of the connection between pregnancy and the production of milk as food for the newborn in humans, but this knowledge was not yet transferred to the context of the cow.

Michael (10), on the other hand, believed that dairy cows produce milk before the first calving and that milk production is enhanced by a pregnancy and calving. At the same time, he emphasized that milk is produced for the calf and that the calf is deprived of milk when its mother is milked. This suggests that Michael (10) could not identify any interdependence between pregnancy/calving and milk production. Thus, the awareness that a cow produces milk for her calf is not alone sufficient to trigger a cognitive conflict regarding milk production prior to first calving (50).

All students except Mark (6) imagined that a cow on a dairy farm is naturally inseminated. Students' conception of natural insemination on dairy farms explains why almost all of the students imagined at least one bull on a dairy farm in addition to dairy cows. This romanticized conception does not correspond to the reality on dairy farms, as the majority of conventional dairy farms and even 80–90% of organic dairy farms in Germany use artificial insemination (33, 34). Eva (10) and Jürgen (10) showed more elaborated conceptions of insemination on dairy farms. For example, Jürgen (10) described artificial insemination on dairy farms as a process in which genetic materials are inserted into the cow. However, he assumed that the genetic materials for insemination could also be ingested through feed. He might have derived this unrealistic conception from his knowledge about special feed for increasing milk yield.

Mark (6) was the only student who excluded natural insemination on dairy farms. He even named an insemination technician in the context of insemination on dairy farms, which is consistent with the real-life approach (31, 33).

#### 4.3.2. Process of milk production

The students predominantly described the stomach (for nutrient utilization) and the udder as organs involved in lactation. This conception can be classified as realistic, as is the conception that the nutrients in milk are absorbed through food (31).

In contrast, the conceptions about the anatomical location of milk production were largely unrealistic. Anna (6) stated that milk production occurs in the intestines, while Johannes (6) and Kristina (10) assumed that it occurs in one of the cow's stomachs. Marie (6) and Jürgen (10) believed that milk production occurred in an unspecified separate organ in front of the udder. Accordingly, for a large proportion of the students, the udder functioned only as a “storage location” for the milk, not as a “production location.” In this context, Johannes (6), Marie (6), Kristina (10), and Jürgen (10) also imagined the udder as an empty container after milking. However, since milk formation takes place exclusively in the udder, which consists of mammary gland tissue and complex blood, nervous, and lymphatic systems, among other things (31), it is clear that the majority of the students do not have a basic understanding of the production of milk. Along these lines, only six of the eight students described an udder consisting of four teats. The students' conceptions about the structure of an udder are fundamentally in line with the results from the study by Brämer (39), according to which only 64% of the students knew that a cow's udder has four teats.

Mark (6) was the only student who showed elaborate conceptions of milk formation, describing the udder as the place where milk is produced and, in this context, naming the mammary gland tissue as well as the cisterns. According to Mark's (6) statement, the mammary gland tissue is permanently present in the udder and some milk remains in the udder even after milking. It is likely that Mark's (6) conceptions about the milk formation process are based on his primary experiences on a dairy farm (milking and making dairy products; Table 1).

### 4.4. Comparison of the conceptions of the sixth- and tenth-grade students

When analyzing the students' conceptions of keeping dairy cows, no significant differences could be discerned between the sixth- and tenth-grade students (RQ1, Section 3.1.). Regarding the stock size and the localization of dairy farms in Germany, both the sixth and tenth graders had realistic conceptions (see Section 3.1.1.). The only conspicuous feature was the differentiation of the statements by the tenth graders. Tenth graders overwhelmingly indicated multiple locations where dairy farms are localized. Moreover, they anticipated several stock sizes, while the sixth graders mostly gave only one answer. Regarding pasture grazing, the conceptions of both the sixth and tenth graders were idyllic and romanticized. It can be inferred that for the students, the conception of cows grazing in a pasture was sufficient to explain the production of milk and that neither sixth- nor tenth-grade students had ever needed to construct a new conception of pasture grazing (23, 24).

The conceptions regarding the design of the cowsheds were very detailed and mostly realistic for both the sixth and tenth graders (see Section 3.1.2.). However, negative and partly outdated conceptions about the keeping of dairy cows could be identified in the case of two tenth graders. These results are in line with those of the study by Folsche and Fiebelkorn (38), in which elementary school students realistically described common husbandry practices in dairy farming, although romanticized as well as decidedly negative conceptions could be identified (38). For the two tenth graders, negative and partly outdated conceptions about the design of cowsheds may have been shaped by primary and secondary experiences with dairy farms.

In contrast, the sixth and tenth graders differed in their conceptions of calf husbandry. While the sixth graders had romanticized conceptions of the cohabitation of mother cow and calf after birth, the tenth graders had mainly realistic ideas (see Section 3.1.2.). The romanticized conception that the dairy cow and her calf stay together for a long time after birth may be due to the fact that the sixth graders do not yet associate the birth of a calf with industrialized agriculture and mass production. In this regard, an individualization and personification of the dairy cow and her calf could contribute to this romanticized conception (51).

The conceptions of the sixth and tenth graders about the milking process and milking techniques can also be classified as mostly realistic (RQ2, Section 3.2.). All students were aware that nowadays, the milking process is carried out by machines to increase its effectiveness. In this context, the students had realistic conceptions about milking frequencies and duration. Sixth and tenth graders' conceptions of the milking process and milking technology did not differ significantly. It is clear that the sixth and tenth graders had a basic understanding of industrial agriculture with regard to the technical aspects of the milking process (6).

Sixth and tenth graders' conceptions of the reasons for and process of milk production differed from scientific concepts (RQ3, Section 3.3.). Comparing the stated reasons for milk production among all students, more sixth than tenth graders linked milk production to calving (Section 3.3.1.). The tenth graders were only able to imagine that a cow must have been pregnant at one time to produce milk after a hint from the interviewer. One reason for this could be that in the Lower Saxony curriculum for science education, the topic of livestock farming is only covered until the sixth grade (52). This is also reflected in the secondary experiences of the tenth graders with the topic of dairy farming, as all the students were unsure when they had learned about dairy farming in school and what content was covered (Table 2).

Regarding reproduction on dairy farms, the tenth graders showed more differentiated conceptions than the sixth graders. However, only the sixth grader Mark had realistic and elaborated conceptions about artificial insemination on dairy farms (see Section 3.3.1.). Therefore, it could be assumed that the students do not have a basic knowledge of the quantities of milk produced for consumption in Germany, which results in the need for artificial insemination. In addition, the students may not be aware of the limitations of relying on natural insemination, as this may also be a factor in their unrealistic conceptions of insemination on dairy farms. Awareness of the increased efficiency in milk production with using automated milking systems is apparently not sufficient to trigger a cognitive conflict related to natural insemination on dairy farms (50).

In a more specific consideration of milk production in a cow's body, neither sixth nor tenth graders envisioned the udder as the location of milk production (see Section 3.3.2.). Accordingly, the udder functions only as a “storage place” for them. Where and how milk production takes place was described realistically and in detail only by the sixth grader Mark.

In summary, it can be stated that both the sixth and tenth graders have realistic conceptions regarding dairy farming, the milking process, and milking techniques. By comparing the conceptions of the sixth and tenth graders, it became apparent that the conceptions of the tenth graders were more differentiated and elaborate. For example, the tenth graders gave several possibilities for stock size (see Section 3.1.1.). The more differentiated conceptions of the tenth graders could be due to developmental psychological factors. Adolescents' factual knowledge increases significantly as part of the intelligence domains in the context of cognitive development. In addition, working memory, which is responsible for reasoning, among other things, also makes substantial developmental progress between the ages of five and twenty (53).

The conceptions of the sixth-grader Mark and the tenth-grader Michael are particularly striking: Mark had a particularly well-founded expertise on certain topics for his age, while Michael sometimes had particularly unrealistic conceptions, despite being one of the older students. Their conceptions may be due to the influence of their primary experiences with dairy farms.

### 4.5. Influence of primary and secondary experiences on students' conceptions

The results show that students' primary experiences can significantly influence their conceptions about the production of cow's milk.

With the exception of Michael (10), all students had already been on a farm as part of a class trip or during vacation. Mark (6), Marie (6), Eva (10), and Jürgen (10) had already visited milking plants, and some were even allowed to milk cows themselves. These students had realistic conceptions about the keeping of dairy cows as well as the milking process and the technology used (see Sections 3.1. and 3.2.).

Michael (10) was the only student who had only minor primary experience with farms—he had only had contact with a farm with fattening bulls. The lack of intensity and quality of experiences with dairy farming could be a reason for Michael's unrealistic conceptions about, for example, milk production (48, 49).

The particularly elaborate and detailed conceptions of Mark (6) can be explained by his regular primary experiences on an alpine pasture, where he was allowed to milk cows and make dairy products himself. In addition, he expressed that in his free time he watches knowledge programs on agriculture topics. Thus, active primary experiences with dairy farms can contribute to improved conceptions about dairy farming (48, 49).

The studies by Hamann (37) and Folsche and Fiebelkorn (38) also demonstrated that students' primary experiences can have a major impact on their conceptions of agriculture.

In contrast, it can be assumed that students' secondary experiences play a rather minor role in their mental constructs. Agricultural topics, such as livestock farming, are covered in science lessons in Lower Saxony once in elementary school and once at the beginning of high school (52, 54). The tenth graders had only vague recollections of the treatment of agricultural topics in science classes (Table 2). In comparison, the sixth graders could remember their secondary experiences more concretely (Table 1). Therefore, it can be hypothesized that secondary experiences with dairy farms have an impact on students' conceptions only shortly after the experience.

## 5. Implications for education and research

The results of this study show that, depending on their age and experience with dairy farms, students' conceptions can vary significantly from idyllic, romanticized conceptions to detailed and elaborated conceptions of dairy farming and milk production. Moreover, even if students grow up in a region with intensive dairy cow husbandry, primary and secondary school teachers should not assume that students have realistic conceptions of dairy farming and milk production. To promote students' understanding of the production and consumption of animal products like milk, an important component of promoting responsible consumption and production of food (SDG 12), the stock size and husbandry of dairy cows as well as the process of milk production can be crucial information to include in lesson design (17, 38). For example, the relationships between the amount of milk produced on a dairy farm in Germany, the development of herd size, dairy cow husbandry, and the process of milk production could be addressed in class. This is also a suitable context in which to highlight the social (SDG 1, 3), ecological (SDG 6), and economic aspects of livestock production that affect the sustainable production of our food in the context of ESD (SDG 12) (17, 55).

Here, we present an example of how to use student conceptions of dairy farming and milk production to structure teaching lessons to stimulate the reconstruction of students' conceptions. Notably, Posner et al. (24) listed dissatisfaction with the existing conception and logic, plausibility, and fruitfulness of the new conception as conditions for changing pre-instructional conceptions. Michael's (10) conceptions of the milking process, milking frequency, and milk production provide a good starting point for reconstruction. For example, his conceptions of the milking process and milking frequency could be contrasted with the amount of milk produced annually in Germany. Michael could first explain why he believes that a cow is milked in the stall and only several times a month. Based on this conception, students could be asked to assess whether an annual production volume of 33 million tons of cow's milk is feasible under these circumstances (6). For this purpose, the students could be given the number of dairy cows in Germany and the amount of milk that a cow produces per day as further information. Subsequently, the teacher could lead a discussion on how a cow produces 25 liters of milk per day. This would also make the connection between food production and consumption patterns in terms of ESD (17). To show the interdisciplinarity of the topic, the global demand for milk in view of the growing world population, factory farming and the impact on the environment could be discussed in the context of SDG 2, 6, and 12.

As demonstrated in previous studies, primary experiences with dairy farming and dairy cattle can influence students' conceptions (37, 38, 48, 49). To counteract the romanticized conceptions—which exist among both sixth and tenth graders—field trips should be designed to provide direct experiences with dairy farms. To ensure that field trips do not present a limited picture of agriculture, the teacher should provide appropriate preparation and follow-up. Previous studies have demonstrated that real-life encounters with farms have a positive effect on interest in agricultural topics and promote realistic conceptions of farm animal husbandry (48, 49).

In this study, it was found that secondary experiences tended to play a minor role in students' conceptions. In this context, the study by Schütte and Busch (41) demonstrated that only half of primary and secondary teachers in Germany teach agricultural topics on a regular basis. In addition to differences in the regularity of teaching series related to agriculture, the way agricultural topics are taught in the classroom may vary (40). Therefore, it is not possible to derive a generalized statement about the influence of secondary experiences with dairy farms on students' conceptions. However, regularly conducting farm-related lesson series can be promoted among teachers by, for example, developing a lesson series on ESD that promotes students' expertise in farm animal husbandry and sustainability and addresses the social, environmental, and economic aspects of farm animal husbandry for sustainable production of our food.

Due to the small sample size and the qualitative research design, the results of this study are not representative of sixth- and tenth-grade students in Germany. Therefore, to generate valid statements regarding student conceptions of milk production with a focus on dairy farming, milking, and milk formation, one could increase the sample size. If the sample is enlarged, an expansion of the ages/school classes should be considered to obtain representative results on student conceptions of milk production in Germany and the differences across ages/school classes. As a further option, the students' conceptions of milk production could be investigated in a quantitative follow-up survey.

Furthermore, it should be noted that the students' conceptions were evaluated with an interpretative methodology. Therefore, misinterpretations of the students' statements cannot be excluded (25–27). Moreover, this study did not distinguish between the conceptions of students who grew up in the city and those who grew up in the countryside. For a differentiated consideration of the influence of primary experiences, one could take this parameter into account when designing the sample in another study.

## Data availability statement

The raw data supporting the conclusions of this article will be made available by the authors upon request without undue reservation.

## Ethics statement

Ethical review and approval was not required for the study on human participants in accordance with the local legislation and institutional requirements. Written informed consent to participate in this study was provided by the participants' legal guardian/next of kin.

## Author contributions

LS: formal analysis, writing—original draft, and writing—review and editing. FF: conceptualization, writing—review and editing, project administration, resources, and supervision. GO: investigation, data curation, formal analysis, and writing—original draft preparation. LA: investigation, data curation, and writing—original draft preparation. EF: writing—review and editing. All authors contributed to the article and approved the submitted version.
